# Conflicting Roles of ZFP36L1 in Regulating the Progression of Muscle Invasive Bladder Cancer

**DOI:** 10.3389/fmolb.2022.687786

**Published:** 2022-03-11

**Authors:** Simin Yuan, Yujia Zhai, Tao Tao, Xiaolong Zhang, Ghassan Bashir, Guangzhi Li, Gang Wang, Song Wu

**Affiliations:** ^1^ Department of Anesthesiology, The Third Affiliated Hospital of Shenzhen University (Luohu Hospital Group), Shenzhen, China; ^2^ Shenzhen Following Precision Medicine Research Institute (Luohu Hospital Group), Shenzhen, China; ^3^ Department of Urology, The Third Affiliated Hospital of Shenzhen University (Luohu Hospital Group), Shenzhen, China; ^4^ State Key Laboratory of Oncology in South China, Collaborative Innovation Center for Cancer Medicine, Sun Yat-Sen University Cancer Center, Guangzhou, China; ^5^ Medical College, Shenzhen University, Shenzhen, China

**Keywords:** TCGA, invasiveness, self-renewal, muscle invasive bladder cancer, Zfp36l1

## Abstract

As the most common carcinoma of the human urinary system, bladder cancer (BC) is characterized by high recurrence, and poor prognosis after metastasis. In the past decade, genome-wide expression and sequencing studies had identified key genes and pathways related to BC, and pictured the comprehensive molecular features of the disease. Our previous study indicated that the coding gene of zinc finger protein 36 like 1 (*ZFP36L1*) mutated frequently in bladder tumor tissues and may be a potential suppressor for BC. The present study aimed to further investigate the role of *ZFP36L1* in BC, and the survival analysis based on TCGA dataset revealed that high expressing level of *ZFP36L1* associated with poorer prognosis of the patients with muscle invasive bladder cancer (MIBC). The associations of *ZFP36L1* expression to the clinicopathological and molecular biological features also implicated the high level of *ZFP36L1* may related to worse outcomes of patients. Also, GSEA indicated that high expression of *ZFP36L1* significantly associated with enhanced activity of cancer metastasis related pathways. Functions of ZFP36L1 in MIBC were investigated further, and it was found that while ZFP36L1 suppressed the self-renewal of bladder cancer cells, it promoted the invasiveness of the cells markedly. Taken together, these results led to the conflicting roles of ZFP36L1 in regulating the progression of MIBC, and revealed further researches are needed to clarify the functions of the gene in tumor initiation and recurrence.

## Introduction

Bladder cancer (BC) is known to be the most common carcinoma of urinary system, with an estimated 5,49,393 new cases and 1,99,922 deaths worldwide in 2018 alone (Bray et al., 2018). Approximately 75% of the new BC cases were diagnosed to be the early-stage (Ta-T1) non-muscle invasive bladder cancer (NMIBC), which is characterized by a good prognosis (5-years survival >90%) and a strong tendency of recurrence. (Flaig et al., 2020). However, about 10–15% of the NMIBC cases recur to more advanced stage (T2-T4), and should be redefined as muscle-invasive bladder cancer (MIBC) for their malignant extension into the muscle (Knowles and Hurst, 2015). MIBCs have less favorable prognosis (5-years survival <50%) and are common to metastasis (Knowles and Hurst, 2015; Flaig et al., 2020), and 5-years survival for metastatic cases is less than 5% (Knowles and Hurst, 2015; Akaza et al., 2016). Deeper understanding of molecular mechanisms under the progression of MIBC is urgently required for the development of improved treatments.

In a previous study, we had performed a deep whole-genome sequencing of bladder tumor biopsies from 65 individuals, and characterized the mutations associated with BC by comparing the tumor tissues to the matched peripheral blood samples (Wu et al., 2019). Frequent mutations were found in the protein-coding exons of a series known bladder tumorigenesis-related genes, such as *FGFR3*, *TP53, PIK3CA, HRAS, and KDM6A*, etc. *ZFP36L1*, which was included in the OncoKB Cancer Gene List (https://www.oncokb.org/) as a tumor suppressor gene (Chakravarty et al., 2017), mutated in 12.3% BC samples. The mutation types identified in *ZFP36L1* included deletion, frameshift deletion, frameshift insertion, missense mutation, nonsense mutation, and synonymous mutation, most of which tended to damage the structure or function of the gene. Moreover, comparing the gene expression in six BC cell lines to that in an immortalized normal bladder urothelial cell line revealed the lowly expressed *ZFP36L1* in BC cells (Wu et al., 2019). High mutation frequency of *ZFP36L1* (7%) was also confirmed in the 412 BC cases from the Cancer Genome Atlas (TCGA) (Cancer Genome Atlas Research, 2014; Robertson et al., 2017). These results seemed to be proving the tumor-suppressor properties of *ZFP36L1* in BCs as reported in a recent study (Loh et al., 2020).

*ZFP36L1* encodes the protein of Zinc Finger Protein 36 Like 1 (ZFP36L1), which belongs to the tristetraprolin (TTP) protein family. Mechanistically, ZFP36L1 functions by binding to the AU-rich elements within the 3′-untranslated regions of its target mRNAs and thus resulting the decay of the latter (Saini et al., 2020). Germline deletion of Zfp36l1 in mice was embryonically lethal, most of the deaths were caused by the failure of chorioallantoic fusion while the others were likely caused by the decreased cell proliferation within placentas (Stumpo et al., 2004). In the last decade, roles of the TTP family proteins in carcinogenesis have been researched, and abundance of evidence suggests a protective role of the TTP proteins in tumorigenesis. Oncogenes like NOTCH1, NANOG, and BCL-2 were reported to be the targets suppressed by ZFP36L1 in several previous studies (Hodson et al., 2010; Tan and Elowitz, 2014; Zekavati et al., 2014). Downregulation of ZFP36L1 was considered to be associated with some cancers (Chen et al., 2015; Rataj et al., 2019; Loh et al., 2020; Saini et al., 2020). However, upregulation of ZFP36L1 was also proved to be related to the initiation and progression of cancers. For instance, ZFP36L1 was found overexpressed in lymph node positive primary breast tumors, and required for the gliomagenesis (Abba et al., 2007; Weng et al., 2019). The contradiction among the evidences reveals that conclusions about the role of ZFP36L1 in carcinogenesis should be made more carefully.

In the current study, we aimed to gain insight on the function of *ZFP36L1* in bladder cancer and explore the correlation between *ZFP36L1* expression and the prognosis of BC patients. The results suggest that *ZFP36L1* may play opposite roles in the self-renewal and invasion of BC cells respectively.

## Results

### The Expression of *ZFP36L1* in MIBCs Compared to Normal Tissues

**FIGURE 1:**
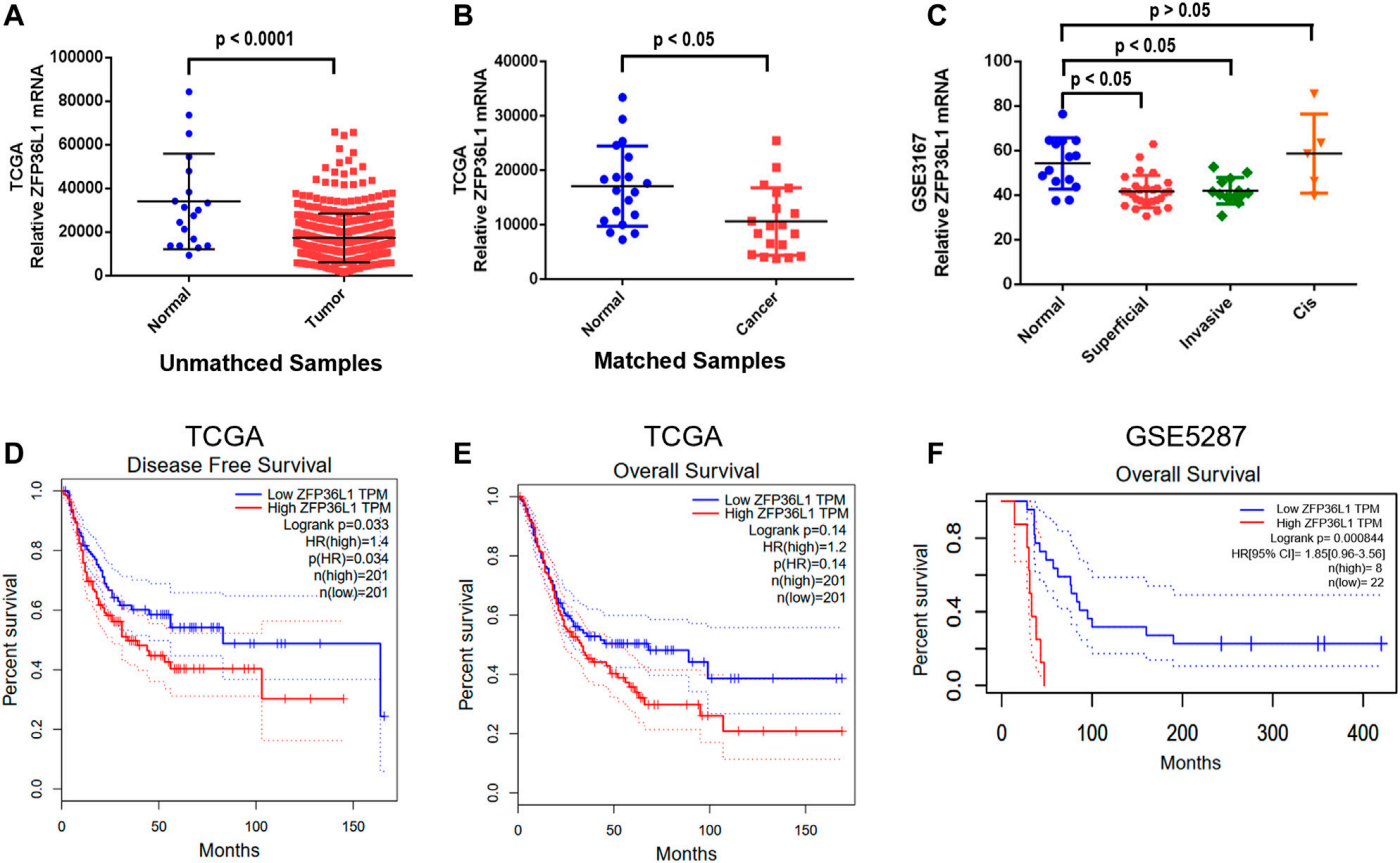
Expression of *ZFP36L1* in different bladder cancer specimens and the correlation analysis of survival time with the expression level. **(A)** Comparison of the transcription of *ZFP36L1* between the 408 bladder tumors and 19 bladder normal tissues from the database of TCGA; **(B)** Comparison of the transcription of *ZFP36L1* exclusively between the 19 paired matched samples of bladder tumor and their adjacent normal tissues in TCGA; **(C)** Comparison of the transcription of *ZFP36L1* among superficial bladder tumors (*n* = 28), invasive bladder tumors (n = 13), CIS (*n* = 5), and normal bladder tissues (*n* = 14) from the transcriptome profiling array of bladder carcinoma contributed by Dyrskjøt L *et al* in GEO database (GSE3167). Disease free survival **(D)** and overall survival **(E,F)** analysis of ZFP36L1 with the muscle invasive bladder cancer transcriptome data of TCGA and GSE5287 predicting the correlation between the expression of the gene and the prognosis of the patients with MIBC.

To investigate the role of ZFP36L1 in MIBCs, we analyzed the *ZFP36L1* expression in the MIBC cohort (427 samples) obtained from the TCGA. As shown in Figure 1A, the expression of *ZFP36L1* is significantly lower in tumor than that in normal tissues among the unmatched MIBC samples in TCGA (408 tumors vs. 19 normal tissues; *p* < 0.0001, Wilcoxon rank sum test). Comparison exclusively between the 19 paired matched samples of tumor and their adjacent normal tissues in TCGA also indicated the low expression of *ZFP36L1* in MIBC, even with a less significant difference (*p* < 0.05, Paired Student’s t-test) (Figure 1B). Then we derived the expression data of *ZFP36L1* from the transcriptome profiling array of bladder carcinoma contributed by Dyrskjot L *et al* in GEO database (GSE3167). The result suggested the significant down-regulation of *ZFP36L1* in superficial and muscle-invasive bladder cancer (MIBC), respectively, and but not in the carcinoma *in situ* (CIS) (Figure 1C). Altogether, decreased *ZFP36L1* expression in MIBCs compared to normal urothelium tissues was confirmed in our analysis.

### High-Expressed *ZFP36L1* Suggests Worse Prognosis of MIBCs

To clarify the potential correlation of ZFP36L1 with the prognostics of the patients with MIBC, survival analysis was performed based on the expression data from TCGA and GSE5287. Interestingly, the *ZFP36L1* ^High^ patients had significantly lower disease-free survival rate (*p* < 0.05, Log-rank test) (Figure 1D) and lower overall-survival rate (*p* < 0.05, Log-rank test, based on the GSE5287 dataset) although not significantly in the TCGA dataset (Figures 1E,F). These results seemed to be contradicted with the tumor suppressor role of ZFP36L1 in bladder cancer suggested by previous studies.

### The Associations of *ZFP36L1* Expression to the Clinicopathological and Molecular Biological Features of MIBC

**FIGURE 2:**
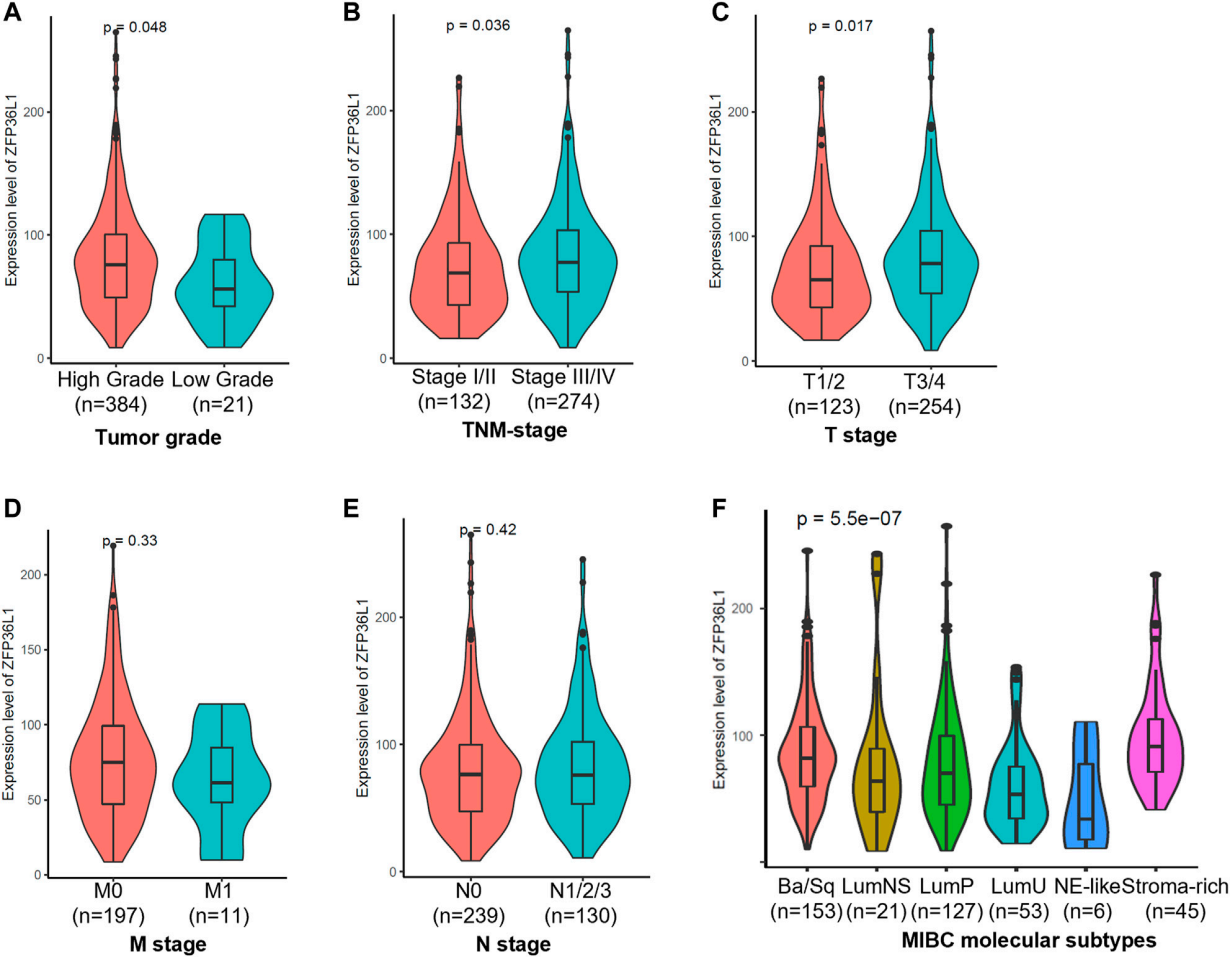
Analysis of the associations of *ZFP36L1* expression to the clinicopathological and molecular biological features of TCGA-BLCA patients. Comparison of the ZFP36L1 expression in different **(A)** tumor grades, **(B)** TNM (Tumor node metastasis) stages, **(C)** T (Tumor) stages, **(D)** M (Metastasis) stages, **(E)** N (Node) stages, and **(F)** molecular subtypes of MIBC. *p* < 0.05 was recognized as statistically significant.

To gain a comprehensive understanding about the expression of *ZFP36L1* in MIBC tumors of different clinicopathological features, we then investigated the relationships between *ZFP36L1* expressing levels and clinicopathological parameters in the TCGA-BLCA patients. The expression of ZFP36L1 was significantly higher in patients with a high-grade tumor (*p =*0.048 < 0.05) (Figure 2A), a high-TNM stage tumor (*p* = 0.036 < 0.05) (Figure 2B), and a high-tumor (T) stage tumor (*p* = 0.017 < 0.05) (Figure 2C), but showed no association with metastasis (M) stage (*p* = 0.33) and node (N) stage (*p* = 0.42) (Figures 2D,E).

To achieve an international consensus on MIBC molecular subtypes that reconciled the published classification schemes, Aure´lie Kamouna *et al* had identified a consensus set of six MIBC molecular subtypes based on 1750 MIBC transcriptomic profiles from 16 published datasets and two additional cohorts (Kamoun et al., 2020). These six subtypes, including Ba/Sq (basal/squamous), LumP (luminal papillary), LumNS (luminal nonspecified), LumU (luminal unstable), NE-like (neuroendocrine-like), and stroma-rich, differ regarding underlying oncogenic mechanisms, infiltration by immune and stromal cells, and histological and clinical characteristics. To investigate the expressions of *ZFP36L1* in MIBCs of different molecular biological characteristics, we compared the expression levels of ZFP36L1 in the 6 subtypes of TCGA-BLCA patients (*p* = 5.5e-07, Kruskal–Wallis test) (Figure 2F; Supplementary Table S1). And the result showed that compared to in the subtype of LumP, the expressions of ZFP36L1 were significant higher in the subtypes of Ba/Sq (*p* = 0.015 < 0.05), LumU (*p* = 0.006 < 0.05), and stroma-rich (*p* = 0.001 < 0.05). The differences of ZFP36L1 expressions between the LumP and LumNS (*p* = 0.52) or NE-like (*p* = 0.1) were not significant. According to the report, while LumP tumors were characterized by high expression of a noninvasive Ta pathway signature, LumU tumors had a higher cell cycle activity. The Ba/Sq and stroma-rich subtypes were both characterized by immune infiltration, while the former was also associated hypoxic microenvironment (Kamoun et al., 2020).

### High Expression of *ZFP36L1* Is Associated With Enhanced Activity of Cancer Metastasis Related Pathways

**FIGURE 3:**
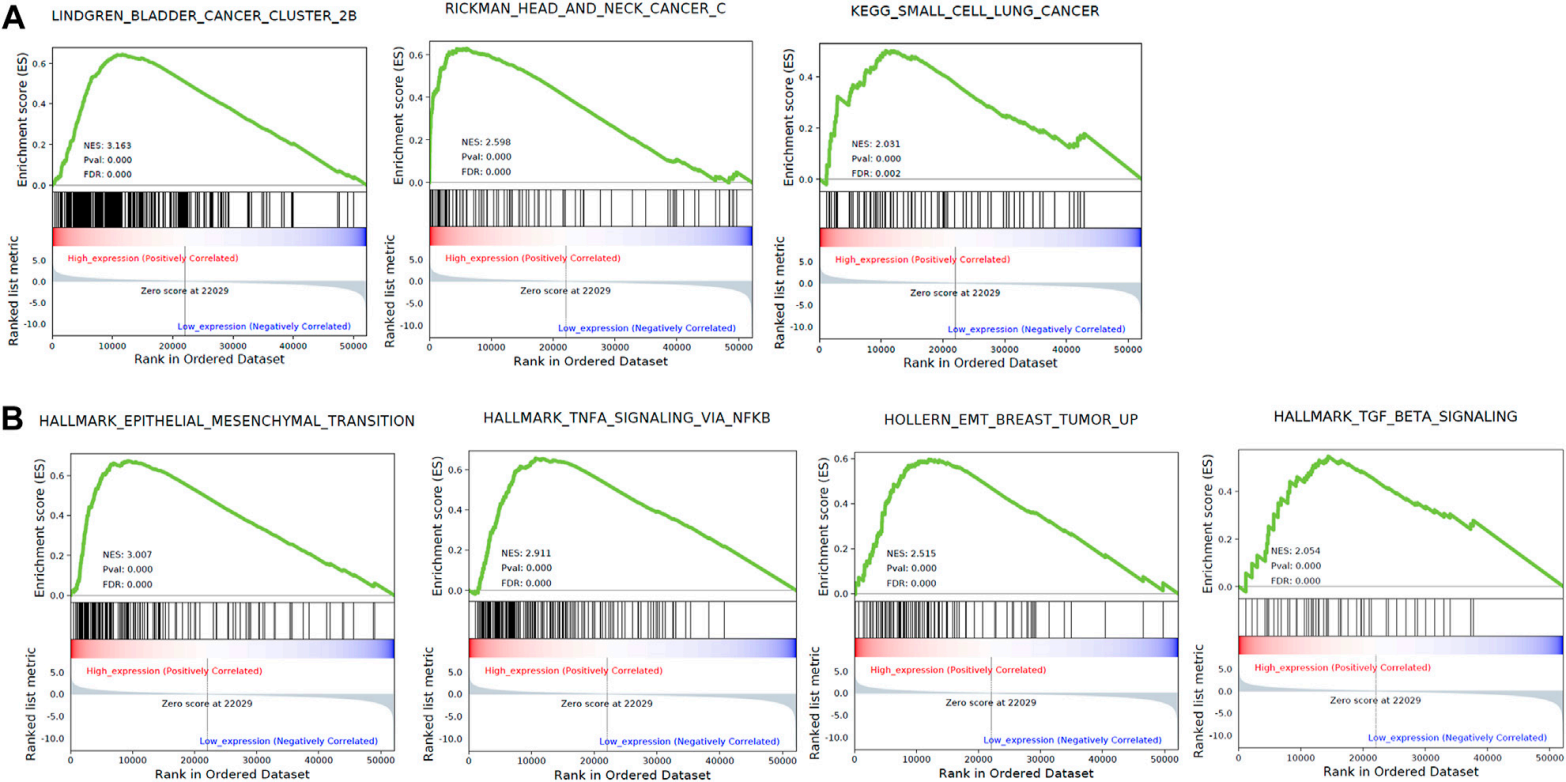
GSEA based on the TCGA bladder cancer data identified 3 cancer associated gene sets **(A)** and 5 metastasis associated gene sets **(B)** enriched in the ZFP36L1 high expressing group.

**FIGURE 4:**
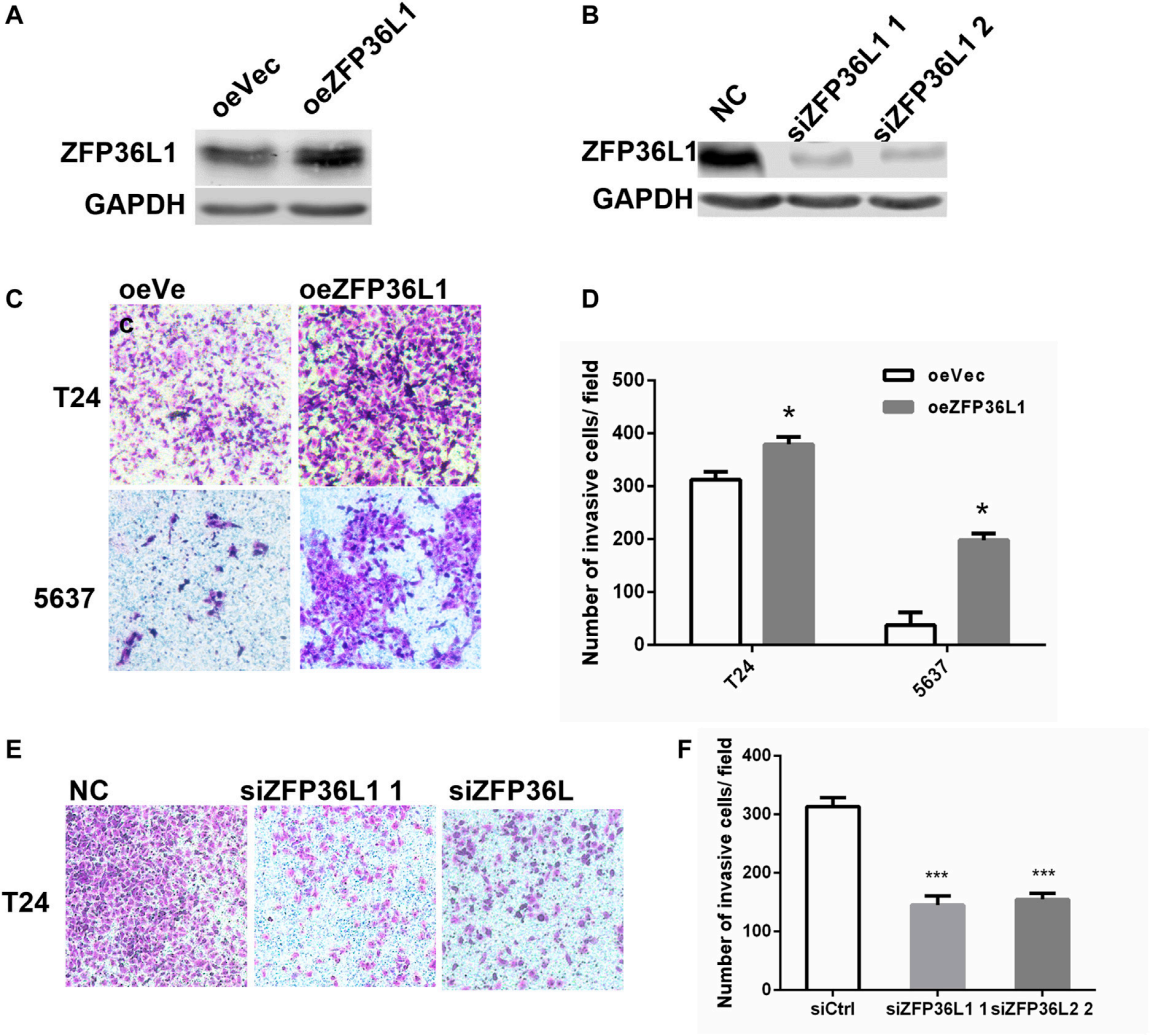
Invasiveness promoting effect of ZFP36L1 on MIBCs. **(A)** Expression level of ZFP36L1 in the ZFP36L1-overexpressing bladder cancer cells (oeZFP36L1) compared to the control cells (oeVec); **(B)** Expression levels of ZFP36L1 in the ZFP36L1-knocked down bladder cancer cells (siZFP36L1 1 and siZFP36L1 2) compared to the control cells (NC); **(C,D)** Overexpression of ZFP36L1 promoted the invasiveness of the T24 and 5,637 bladder cancer cells; **(E,F)** Knocking down the ZFP36L1 with siRNA suppressed the invasiveness of the T24 bladder cancer cells significantly.

To investigate the biology function of ZFP36L1 during cancer progression, we separated MIBCs from TCGA as *ZFP36L1* ^High^ and *ZFP36L1* ^low^ basing on the expression level of *ZFP36L1* (top and bottom quartiles, respectively), and performed Gene Set Enrichment Analysis (GSEA) between these two subgroups. In this process, five databases including GO, KEGG, Reactome, CGP, and Hallmark, were included for enrichment analysis. 1,298 gene sets are significantly correlated with the expressions of *ZFP36L1* (FDR <0.05) (Supplementary Table S2). For each database, the top 10 significantly enriched gene sets (defined and sorted by the value of FDR) were considered affected by the expression level of *ZFP36L1* remarkably and were depicted further (Supplementary Figure S1). All these 60 gene sets were identified in the ZFP36L1^High^ > ZFP36L1^Low^ analysis (NES >0). Notably, 3 cancer associated gene sets (LINDGREN_BLADDER_CANCER_CLUSTER_2B, RICKMAN_HEAD_AND_NECK_CANCER_C, and KEGG_SMALL_CELL_LUNG_CANCER) (Figure 3A) and 4 metastasis gene sets (HALLMARK_EPITHELIAL_MESENCHYMAL_TRANSITION, HALLMARK_TNFA_SIGNALING_VIA_NFKB, HOLLERN_EMT_BREAST_TUMOR_UP, and HALLMARK_TGF_BETA_SIGNALING) (Figure 3B) are enriched in the *ZFP36L1* highly expressed group. Even though previous studies mostly suggested *ZFP36L1* as a tumor-suppressor gene, the results of survival analysis and GSEA assay above raise the attention to the potential cancer promoting effect of ZFP36L1 in MIBCs.

### Invasiveness Promoting Effect of ZFP36L1 on MIBCs

To examine the metastasis related function of ZFP36L1 in MIBCs, we sought to assess the effect of ZFP36L1 on the invasion of bladder cancer cell lines established from MIBC *in vitro*. Stable overexpression of ZFP36L1 was achieved by transfecting the T24 and 5,637 cells with the ZFP36L1 harboring-lentivirus and confirmed by western blotting assay (Figure 4A). Matrigel-coated transwell assays were performed and showed that the ZFP36L1 overexpressing cells presented a higher capability of invasion compared to the control (Figures 4C,D). Complementary experiments were performed with the *ZFP36L1*-knocking down cells constructed by siRNAs (Figure 4B). As shown in Figures 4E,F, reduced ZFP36L1 attenuated the invasiveness of bladder cancer cells.

### ZFP36L1 Suppresses the Self-Renewal of MIBC Cells

**FIGURE 5:**
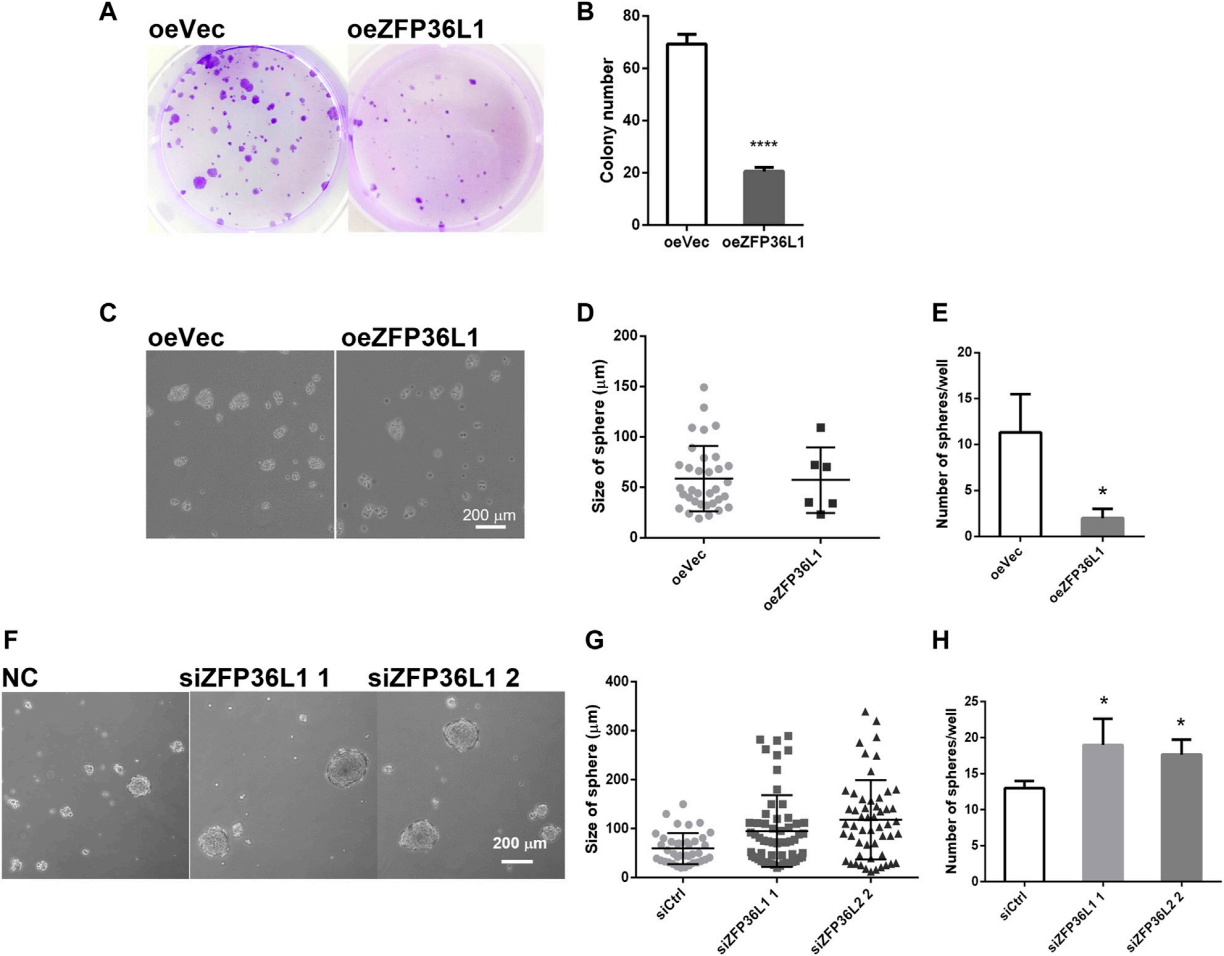
ZFP36L1 suppresses the self-renewal of MIBC cells. **(A,B)** Overexpression of ZFP36L1 inhibited the colony-formation capability of bladder cancer cells; **(C–E)** Overexpression of ZFP36L1 inhibited the sphere-formation capability of bladder cancer cells. *p < 0.05 and ****p < 0.0001. Data are representative of three independent experiments; **(F–H)** ZFP36L1 knocking down enhanced the sphere-formation capability of bladder cancer cells significantly. *p < 0.05 and ***p < 0.001. Data are representative of three independent experiments.

The regulation on the growth of MIBC cells by ZFP36L1 was then investigated in the *in vitro* system of this study. Overexpression of ZFP36L1 markedly repressed the proliferation of bladder cancer cells as shown in the colony formation assay (Figures 5A,B). Furthermore, in the oncosphere formation assay, the sphere forming efficiency of ZFP36L1 overexpressed cells is significantly lower than the control cells (Figures 5C–E), while *ZFP36L1* knockdown increases both the quantity and size of the oncospheres formed by the same number of cells (Figures 5F–H). To sum up, corresponding to previous studies, ZFP36L1 played as a suppressor on the self-renewal ability of MIBC cells.

## Discussion

While newly detected BC cases are commonly NMIBCs and usually followed by good prognosis, patients first diagnosed with or progressed to MIBC may have to accept cystectomy and followed by a high risk of relapse. Metastasis may occur in the relapse when the MIBC cells invade the lymphatic and blood vessels, and the 5 years-survival for these patients is only about 5%. Therefore, it is important to investigate the invasion mechanisms of cancer cells for prolonging the survival time of BC patients.

In the present study, we showed that even though the expression of *ZFP36L1* is lower in BC tumor cells than in normal bladder cells, the high expressing level of *ZFP36L1* is associated with worse prognosis of BC patients. In the comprehensive analysis of the associations of ZFP36L1 expression to the clinicopathological and molecular biological features of MIBC, we found high-grade and high-stage tumors expressing higher levels of ZFP36L1 than low-grade and low-stage tumors. Also, high expression of ZFP36L1 were found in the MIBC subtypes of Ba/Sq and LumU, which were reported associated with poorer prognosis of patients (Kamoun et al., 2020). Moreover, GSEA based on the bladder cancer RNA-Seq data identified 5 cancer metastasis associated pathways may be activated by the high level of ZFP36L1. While the tumor-suppressing function of ZFP36L1 was widely reported, results of the survival analysis and GSEA in BC above are needed to be further investigated. By *in vitro* experiments, ZFP36L1 was shown inhibiting the colony formation and oncosphere formation capability of BC cells while promoting their invasion capability. These results combined suggested the tumor initiation suppressing role as well as the tumor invasion promoting role of ZFP36L1 in bladder cancer.

Cell proliferation and invasion are two uncoupled processes during cancer progression, they were proved to be mutually exclusive in certain cellular models, and highlighting dynamics in cell plasticity (Gao et al., 2005). Comparison of the gene expression signatures between the proliferative and invasive melanoma cells showed two different transcription programs (Hoek et al., 2008). In basal cell carcinoma, infiltrative tumor cells do not actively proliferate, and a functional p16^INK4a^-cyclin D-Rb pathway tended to activate the invasion while cease proliferation (Svensson et al., 2003). Previous studies, involving cellular models of bladder cancer and prostate cancer, had shown that the EMT increases the invasion capability of cancer cells but non-EMT cells are more likely to establish new colonies (Tsuji et al., 2008; Celià-Terrassa et al., 2012). In our study, GSEA based on the expression profile of BC revealed that high-expressing ZFP36L1 enhanced the EMT associated pathways (HALLMARK_EPITHELIAL_MESENCHYMAL_TRANSITION, HALLMARK_TNFA_SIGNALING_VIA_NFKB, HOLLERN_EMT_BREAST_TUMOR_UP, and HALLMARK_TGF_BETA_SIGNALING) (Figure 2). Therefore, we reckoned that high level of ZFP36L1 may suppress the self-renewal capability of BC while promote the invasion capability of the cells by activating the EMT associated pathways. Notably, it was reported that expression of ZFP36L1 is induced by abroad variety of growth factors including TGFβ and TNFα, and responses to scratch-wounding in keratinocytes *in vitro* (Hacker et al., 2010). It is still pending to distinguish the high expression of ZFP36L1 is a cause or effect of the EMT of bladder cancer cells *in vivo*.

## Conclusion

This study reveals a potential invasion promoting role of ZFP36L1 in bladder cancer the first time, which may explain the association of ZFP36L1 expression with the prognosis of BC patients somehow. Although limitations of this study, including a lack of *in vivo* experiments evidence, suggest further research is expected, we believe the results above raise attention to the tumor progression regulating role of ZFP36L1 in bladder cancer which may need to be reconsidered more carefully.

## Methods and Materials

### Data Acquisition and Bioinformatics Analysis

TCGA normalized RNA sequencing (RNA-Seq) data sets with estimation of Fragments Per Kilobase of transcript per Million mapped reads (FPKM) and the clinicopathological characteristics information (Supplementary Table S3) from 408 tumor samples and 19 non-tumor samples were retrieved from UCSC Xena (https://xenabrowser.net/datapages/) for further analysis. Gene expression data in GSE3167 based on the Affymetrix Human Genome U133A Array platform were downloaded from gene expression omnibus (GEO) database (https://www.ncbi.nlm.nih.gov/geo/), including normal bladder tissues (9 samples) superficial transitional cell carcinoma with surrounding CIS (13 samples), without surrounding CIS lesions (15 samples), and in muscle invasive carcinomas (13 samples). Kastle–Meyer survival analysis based on the TCGA BLCA dataset and the GSE5287 dataset was performed using the web server named GEPIA (http://gepia.cancer-pku.cn/index.html) and PrognoScan (http://dna00.bio.kyutech.ac.jp/PrognoScan/), respectively. BLCA RNA-seq data was derived from TCGA and the samples were sorted into the top and bottom quartiles of *ZFP36L1* expression (ZFP36L1^High^ and ZFP36L1^Low^, respectively). Gene set enrichment analysis (GSEA) was performed via GSEApy (https://github.com/zqfang/GSEApy). We downloaded the terms of Gene Ontology (GO), Kyoto Encyclopedia of Genes and Genomes (KEGG), Reactome, and MSigDB Hallmark pathways, and conducted the GSEA analysis, respectively. The significant gene terms were considered as significantly enriched as false discovery rate (FDR) < 0.05.

### Cells and Culture

Human bladder cancer cell lines 5,637 and T24 were obtained from the American Type Culture Collection (ATCC). The cells were cultured in Roswell Park Memorial Institute (RPMI) 1,640 medium (Gibco, Grand Island, NY) containing 10% fetal bovine serum (FBS), 100 U/mL penicillin, and 100 mg/ml streptomycin (Gibco, Grand Island, NY) at 37°C in a humidified atmosphere with 5% CO_2_.

### Generation of Stable Cell Lines With *ZFP36L1* Overexpression

The coding sequence of *ZFP36L1* (1,017 bp) was amplified from the total cDNA library of the immortalized normal bladder cell line SV-HUC-1, with the primers h-ZFP36L1-F (GAG​GAT​CTA​TTT​CCG​GTG​AAT​TCG​CCA​CCA​TGA​CCA​CCA​CCC​TCG​TGT) and h-ZFP36L1-R (TAG​TCA​CTT​AAG​CTT​GGT​ACC​GAG​GAT​CCG​TCA​TCT​GAG​ATG​GAA​AGT). The fragments then were cloned into the lentivirus plasmid vector pHBLV-CMV-MCS-3FLAG-EF1-ZsGreen-T2A-PURO (Hanbio Biotechnology Co.,Ltd). For virus generation, we transfected the 293T cells with the plasmid constructed above, along with package plasmids (pSPAX2 and pMD2G). Bladder cancer cell lines 5,637 and T24 were transfected by virus supernatants and then screened for the positive clones with 1–1.5 μg/ml puromycin. The stable ZFP36L1 overexpressing cells established then were used in following experiments.

### Knock Down *ZFP36L1* With siRNA in Bladder Cancer Cell Lines

3 types of siRNA oligos against the mRNA of *ZFP36L1* were designed and purchased from GenePharma Co.,Ltd. T24 cells were transformed with the oligos using Lipofectamine RNAiMAX (Invitrogen, Thermo Fisher Scientific, Inc.), according to the manufacturers’ instructions. 18 h later, the total RNAs of the transformed cells were examined by RT-PCR to detect the *ZFP36L1* knocking down efficiency. 2 efficient oligos were chose to be used in further experiments and their sequences were as follows: h-ZFP36L1-siRNA #1: sense 5′-CCC​UCG​UGU​CUG​CCA​CCA​UTT-3′, and antisense 5′-AUG​GUG​GCA​GAC​ACG​AGG​GTT-3’; h-ZFP36L1-siRNA #2: sense 5′- GGA​GGC​ACU​CAG​UCA​CCC​UTT-3′, and antisense 5′- AGG​GUG​ACU​GAG​UGC​CUC​CTT-3’.

### Transwell Invasion Assay

For transwell invasion assay, the upper membrane of the chamber inserts of transwell (8 μm pore size; Corning Costar, Lowell, MA, United States) were precoated with 50 μl of 100 μg/ml Matrigel (BD Biosciences San Diego, CA, United States). Indicated cells (1 × 10^4^/chamber) were serum-starved for 6h first, and then were suspended in 100 μl FBS-free RPMI 1640 medium before be seeded into the upper chamber insert held in the lower well with 600 μl RPMI 1640 containing 20% FBS of the 24-well companion plate. After 24 h of incubation, invading cells at the bottom of the membrane filters were fixed in 4% paraformaldehyde and stained with 0.025% crystal violet, then were photographed under a light microscope. The experiment was repeated 3 separate times, and the number of invading cells was quantified by counting 5 fields in each chamber.

### Oncosphere Formation Assay

5,000 indicated cells were cultured with Knock-Out DMEM/F-12 medium (Gibco, 12,660,012) supplemented with 20 ng/ml EGF (Invitrogen, PHG0314), 20 ng/ml bFGF (Invitrogen, 13256029), 1% N2 (Gibco, 17,502,048), and 2% B27 (Gibco, 17,504,044), in the 6 well ultra-low attachment culture dishes (Corning Costar, Lowell, MA, United States). 10 days later, spheres were counted and photographed under a light microscope.

### Colony Formation Assay

The indicated cells were seeded to 6-well plates (500 cells/well) and cultured with complete medium. After 10 days incubation, the colonies formed were fixed with 4% paraformaldehyde and stained with 0.025% crystal violet solution, then were photographed under a light microscope. The experiment was repeated 3 separate times, and the number of colonies was quantified.

### Statistical Analyses

The GraphPad Prism V6 software and R software v4.0.3 were used for data analysis. Differences between two groups of variables were compared using the two tailed unpaired t-test for variables with normal distributions, and the rank sum test for variables not normally distributed. And Kruskal–Wallis test was used to compare more than two groups of variables. *p* < 0.05 was considered statistically significant.

## Data Availability

The datasets presented in this study can be found in online repositories. The names of the repository/repositories and accession number(s) can be found in the article/Supplementary Material.
