# Supplementary material for: Conflicting Roles of ZFP36L1 in Regulating the Progression of Muscle Invasive Bladder Cancer
Source: Front Mol Biosci. 2022 Mar 11;9:687786. doi: 10.3389/fmolb.2022.687786 (PMC8962643; doi:10.3389/fmolb.2022.687786)
Supplement: Supplementary file 3 [file DataSheet1.PDF]

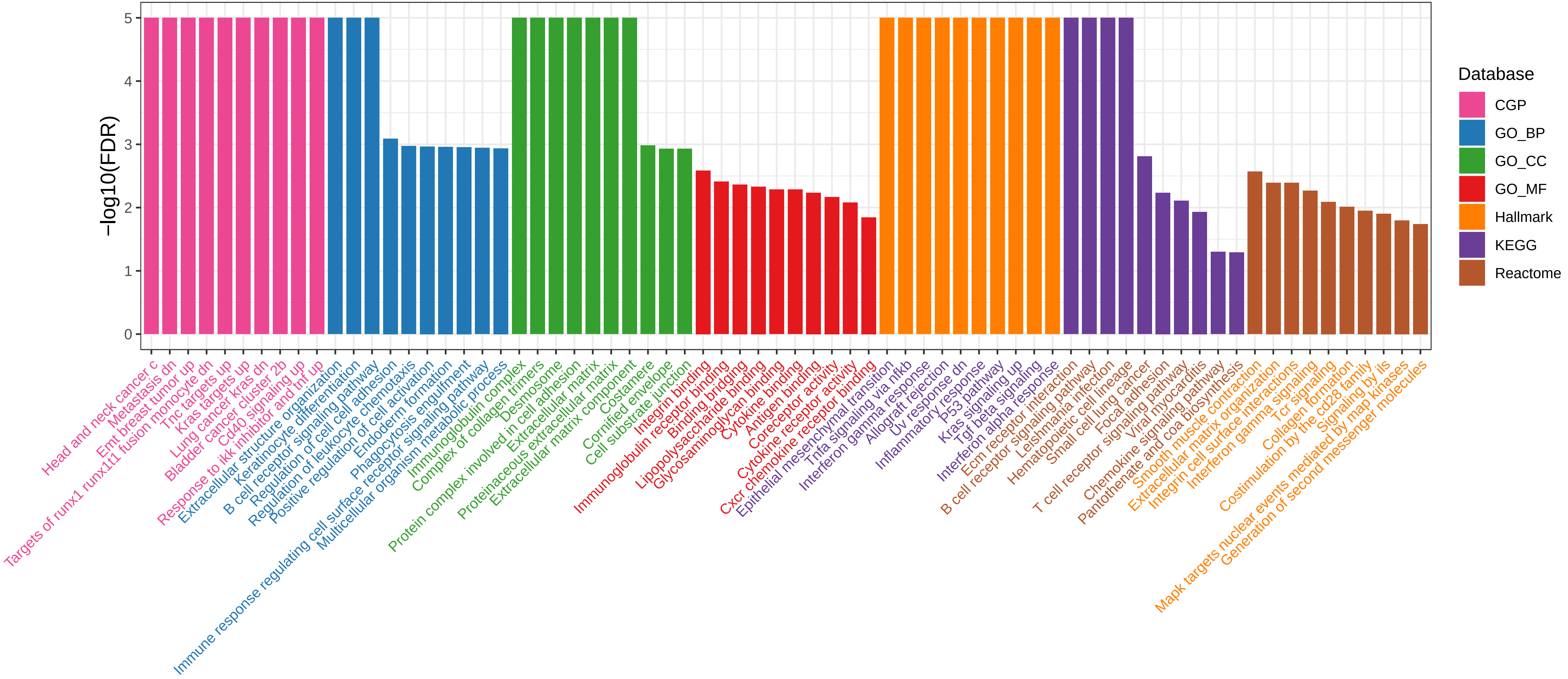

−log10(FDR)
0
1
2
3
4
5
Head and neck cancer c
Metastasis dn
Emt breast tumor up
Targets of runx1 runx1t1 fusion monocyte dn
Tnc targets up
Kras targets up
Lung cancer kras dn
Bladder cancer cluster 2b
Cd40 signaling up
Response to ikk inhibitor and tnf up
Extracellular structure organization
Keratinocyte differentiation
B cell receptor signaling pathway
Regulation of cell cell adhesion
Regulation of leukocyte chemotaxis
Positive regulation of cell activation
Endoderm formation
Phagocytosis engulfment
Immune response regulating cell surface receptor signaling pathway
Multicellular organism metabolic process
Immunoglobulin complex
Complex of collagen trimers
Protein complex involved in cell adhesion
Desmosome
Extracellular matrix
Proteinaceous extracellular matrix
Extracellular matrix component
Costamere
Cornified envelope
Cell substrate junction
Integrin binding
Immunoglobulin receptor binding
Binding bridging
Lipopolysaccharide binding
Glycosaminoglycan binding
Cytokine binding
Antigen binding
Coreceptor activity
Cytokine receptor activity
Cxcr chemokine receptor binding
Epithelial mesenchymal transition
Tnfa signaling via nfkb
Interferon gamma response
Allograft rejection
Uv response dn
Inflammatory response
P53 pathway
Kras signaling up
Tgf beta signaling
Interferon alpha response
Ecm receptor interaction
B cell receptor signaling pathway
Leishmania infection
Hematopoietic cell lineage
Small cell lung cancer
Focal adhesion
T cell receptor signaling pathway
Viral myocarditis
Chemokine signaling pathway
Pantothenate and coa biosynthesis
Smooth muscle contraction
Extracellular matrix organization
Integrin cell surface interactions
Interferon gamma signaling
Tcr signaling
Collagen formation
Costimulation by the cd28 family
Signaling by ils
Mapk targets nuclear events mediated by map kinases
Generation of second messenger molecules
Database
CGP
GO_BP
GO_CC
GO_MF
Hallmark
KEGG
Reactome
